# Psoriasiform adult blaschkitis triggered by adalimumab: a rare paradoxical reaction^؆^

**DOI:** 10.1016/j.abd.2026.501361

**Published:** 2026-05-13

**Authors:** Hiram Larangeira de Almeida, Martina Souilljee Birck, Laura Marcon Bischoff Bialecki, Aline Paganelli, Luísa Becker Martini, Rodrigo Pereira Duquia

**Affiliations:** a Post-Graduation Program in Health, Universidade Católica de Pelotas, Pelotas, RS, Brazil; b Dermatology Service, Santa Casa de Misericórdia de Porto Alegre, Porto Alegre, RS, Brazil; c Pathology Laboratory, Centro de Anatomia Patológica, Pelotas, RS, Brazil; d Graduate Program in Medicine, Universidade Católica de Pelotas, Pelotas, RS, Brazil; e Departamento de Dermatologia, Universidade Federal de Ciências da Saúde de Porto Alegre, Porto Alegre, RS, Brazil

Dear Editor,

Blaschkitis is an acquired, self-limited inflammatory dermatosis that follows Blaschko’s lines, typically affecting adults and involving the trunk. Although its pathogenesis remains unclear, it has been associated with biologic therapies. These paradoxical cutaneous reactions have been reported with Tumor Necrosis Factor Alpha (TNFα) inhibitors such as infliximab, etanercept, certolizumab and adalimumab.^1^, ^2^

A 74-year-old woman with seronegative spondyloarthritis, on adalimumab for three months, developed a gradually progressive, pruritic, erythematous desquamative eruption following Blaschko’s lines on the right trunk (Fig. 1A), with sharp midline demarcation (Fig. 1B). It appeared three months after initiating adalimumab and resolved completely, and without sequelae, following drug discontinuation and substitution to secukinumab. No infectious or other pharmacologic triggers were identified.

**Fig. 1:**
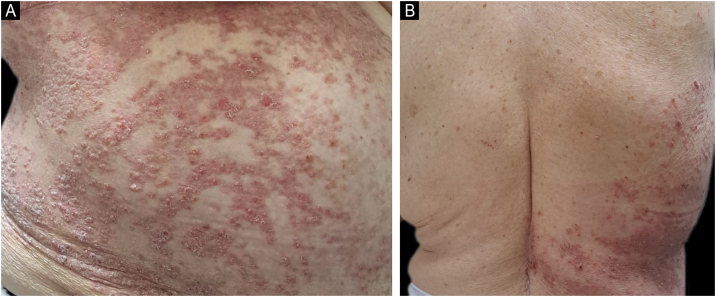
Clinical features. (a) Psoriasiform eruption following Blashko’s lines on the trunk. (b) Midline demarcation on the back.

Histopathology revealed epidermis with psoriasiform hyperplasia with focal thinning of the suprapapillary epidermis, light spongiosis, and mild vacuolar alteration at the interface, as well as acanthosis and focal parakeratosis. There were also aggregates of neutrophils within the stratum corneum. The dermis showed an inflammatory infiltrate composed of lymphocytes, histiocytes, and a few plasma cells around the superficial dermal vasculature, consistent with psoriasiform dermatitis (Fig. 2). Immunohistochemistry was positive in the superficial dermal infiltrate for CD3, CD4 and CD7, whereas CD20 showed negativity (Fig. 3).

**Fig. 2:**
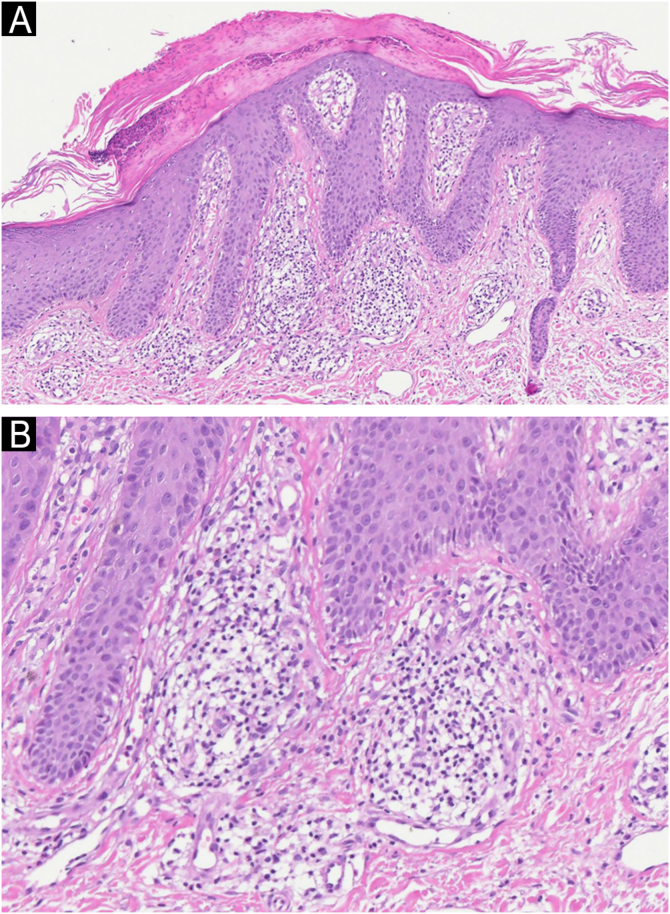
Light microscopy. (A) Psoriasiform hyperplasia with acanthosis and focal parakeratosis, with aggregates of neutrophils within the stratum corneum (Hematoxylin & eosin, ×100). (B) Detail of the dermal infiltrate with lymphocytes and histiocytes (Hematoxylin & eosin, ×200).

**Fig. 3:**
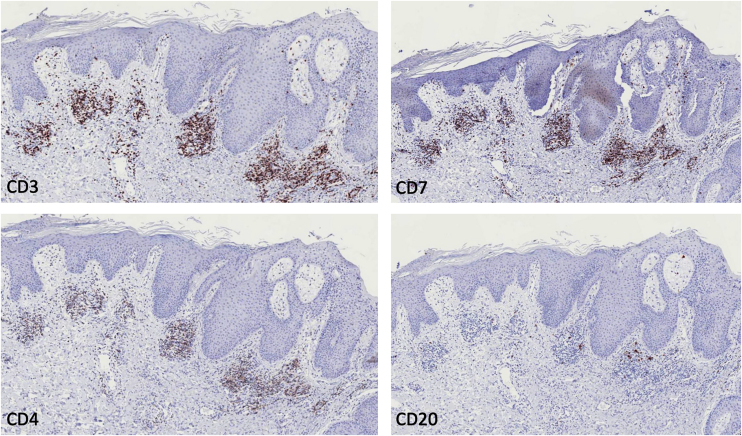
Immunohistochemistry. Positivity in the superficial dermal infiltrate for CD3, CD4 and CD7, and negative for CD20 (×100).

TNFα is a cytokine that plays a pivotal role in the inflammatory response. Its inhibitors have demonstrated significant efficacy in the management of inflammatory diseases such as rheumatoid arthritis, psoriasis, psoriatic arthritis, spondyloarthritis and inflammatory bowel disease. Paradoxically, however, these agents may induce or exacerbate psoriasis.^3^ This phenomenon has been observed across multiple underlying diseases and does not appear to be disease-specific. Moreover, it has been associated with all agents within the anti-TNF class.

A retrospective cohort study compared the risk of psoriasis development among patients with Spondyloarthritis treated with anti-TNF agents versus those receiving conventional therapy. The investigation utilized data from the Korea National Health Insurance Claims Database between January 2007 and December 2016. Psoriatic diseases were identified in 1.8% of patients following initiation of anti-TNF therapy, whereas only 1.1% of patients treated with conventional therapies developed psoriasis.^4^

These findings are further supported by data from a recent meta-analysis.^5^ A statistically higher risk for psoriasis or psoriasiform lesions during anti-TNF therapy was observed in female patients, younger age, smokers, Crohn’s disease, and those who are using adalimumab or certolizumab.

When managing anti-TNF-induced psoriasis or psoriasiform eruptions, clinicians must weigh whether to continue, discontinue, or switch anti-TNF therapy to another drug class. This decision should be individualized, taking into account factors such as the underlying disease, the therapeutic efficacy of the anti-TNF agent, the severity of the cutaneous eruption, and the feasibility of alternative treatment options.

According to the treatment algorithm proposed by Li and Perez-Chada,^3^ the severity of paradoxical psoriasis should first be classified as mild or moderate-to-severe.

For mild cases, if the underlying disease is well controlled, continuation of anti-TNF therapy is recommended, combined with conventional psoriasis treatments (e.g., topical corticosteroids, phototherapy, methotrexate). Furthermore, discontinuation of anti-TNF therapy may have detrimental effects on the control of the underlying inflammatory disease.

When the underlying disease remains uncontrolled despite anti-TNF therapy, switching to another anti-TNF agent combined with conventional psoriasis management may be considered.

In patients presenting with moderate-to-severe eruptions and stable underlying disease, substitution of the anti-TNF agent with a drug from another therapeutic class, in combination or not with conventional psoriasis therapy, is recommended.

If anti-TNF therapy fails to control the underlying condition and paradoxical psoriasis is moderate to severe, switching to an alternative biologic class, along with standard psoriasis treatment, should be considered.

Additionally, similar cases of immunobiologicals-induced psoriasis have been described in patients receiving anti-PD-1 immunotherapy, very likely related to T-lymphocytic infiltration of the skin, as evidenced by our immunohistochemical findings, with positivity to CD3, CD4 and CD7, all T-cell markers.^6^

Some authors have used the denomination Blaschko-linear psoriasis to describe these cases,^7^ the term Blaschkolinear Acquired Inflammatory Skin Eruption (BLAISE) ha salso been used.^8^

The pathogenesis of these paradoxical lesions is not fully understood, very likely, uncontrolled IFN production is released after TNF inhibition, a process driven by the innate immune system, leading to keratinocyte inflammation and proliferation,^9^ our immunohistochemical findings support this, since T-cells are predominant in the dermal infiltrate.

This case highlights psoriasiform blaschkitis as a rare paradoxical dermatologic reaction to TNF-α inhibitors. To date, only one case linked to adalimumab has been reported, making this the second in the literature. Recognizing this self-limited condition,^1^ is essential for appropriate management and to avoid unnecessary discontinuation of effective biologic therapies.

## Authors' contributions

Hiram Larangeira de Almeida Jr: Study concept and design; Data collection, or analysis and interpretation of data; Writing of the manuscript or critical review of important intellectual content; Critical review of the literature, final approval of the final version of the manuscript.

Martina Souilljee Birck: Study concept and design; Data collection, or analysis and interpretation of data; Writing of the manuscript or critical review of important intellectual content; Critical review of the literature, final approval of the final version of the manuscript.

Laura Marcon Bischoff Bialecki: Study concept and design; Data collection, or analysis and interpretation of data; Writing of the manuscript or critical review of important intellectual content; Critical review of the literature, final approval of the final version of the manuscript.

Aline Paganelli: Data collection, or analysis and interpretation of data; Writing of the manuscript or critical review of important intellectual content; Critical review of the literature, final approval of the final version of the manuscript.

Luísa Becker Martini: Data collection, or analysis and interpretation of data; Writing of the manuscript or critical review of important intellectual content; Critical review of the literature, final approval of the final version of the manuscript.

Rodrigo Pereira Duquia: Study concept and design; Data collection, or analysis and interpretation of data; Writing of the manuscript or critical review of important intellectual content; Critical review of the literature, final approval of the final version of the manuscript.

## Financial support

None declared.

## Research data availability

Does not apply.

## Conflicts of interest

None declared.
